# Family Experiences of Living With Frontline Healthcare Workers During the COVID‐19 Pandemic in the United Kingdom: Perspectives From the Black and Asian Community

**DOI:** 10.1155/ipid/5200232

**Published:** 2026-01-13

**Authors:** Ufuomanefe Jones, Amanda Rodrigues Amorim Adegboye, Ranjit Khutan, Moses Murandu

**Affiliations:** ^1^ Research Centre for Healthcare and Communities, Coventry University, Coventry, UK, coventry.ac.uk; ^2^ Public Health Department, University of Liverpool, Liverpool, UK, liv.ac.uk; ^3^ Moses Murandu- Faculty of Education, Health and Well-being, University of Wolverhampton, Wolverhampton, UK, wlv.ac.uk

**Keywords:** Asian and minority ethnic (BAME), Black and Asian, Black, COVID-19 pandemic, family experiences, frontline healthcare workers, healthcare workers, interpretative phenomenological analysis (IPA)

## Abstract

**Background and aim:**

Black and Asian healthcare workers (HCWs) faced heightened COVID‐19 exposure early in the pandemic due to overrepresentation on the frontline compared to other ethnic groups. This study explored the experiences of Black and Asian household members in the United Kingdom living with these HCWs during the COVID‐19 pandemic, focussing on the impact of increased risks and challenges.

**Method:**

This study utilised one‐on‐one interviews with six HCWs’ household members. Participants were recruited through a snowballing sampling strategy. The study employed interpretative phenomenological analysis (IPA) to explore and interpret the meanings participants attributed to their experiences.

**Results:**

The findings highlighted emotional and mental impacts experienced by household members, linked to their ethnic background and living with HCWs. The research found that FMs feared contracting the virus from their HCWs, along with feelings of vulnerability, hopelessness and helplessness. While participants took precautions, they relied on faith and called for specific support, policy changes and reassurance to reduce anxiety.

**Conclusion:**

This study underscores the importance of healthcare organisations addressing the concerns of HCWs from Black and Asian communities and their household members in future pandemics. It recommends establishing platforms within healthcare settings for these workers to voice safety and protection concerns. The study highlights the need for representative channels to listen to their issues and suggests educational seminars to help families manage fears and emotional distress. Ultimately, the research aims to guide policy changes and interventions that offer comprehensive support, ensuring HCWs and their household members are prepared to face health crises with resilience and well‐being.

## 1. Introduction

The COVID‐19 pandemic disproportionately impacted individuals from Black, Asian and minority ethnic backgrounds [1, 2]. In the United Kingdom, evidence revealed higher death rates among Black, Asian and minority populations compared to White groups [1], linked to factors like living in deprived areas, lower‐skilled jobs and pre‐existing conditions such as hypertension and diabetes. Early in the pandemic, Black and Minority healthcare workers (HCWs) faced greater virus exposure due to their overrepresentation on the frontline. The 2021 Office for National Statistics (ONS) analysis during COVID‐19’s second wave found that Black, Asian and minority ethnic groups faced higher risks of severe outcomes than White British groups, even after accounting for employment, living conditions and health factors [3]. This study was limited to Black and Asian because they were the most visible within the Black, Asian and minority ethnic group and were the most hit by the COVID‐19 pandemic.

COVID‐19 significantly impacted HCWs across professions, including nurses, doctors, pharmacists and allied health professionals (AHPs), exposing them to heightened disease risk. Shortages and inadequate personal protective equipment (PPE) further increased health risks, resulting in absences, isolation and transmission risks to household members [4]. Household members in this study are people who are related to the HCWs (genetics or extended). Ethnic groups such as Pakistani, Indian and Bangladeshi were especially vulnerable due to multigenerational living arrangements, which made shielding and protection difficult [5]. Additionally, people from these groups often worked in frontline health roles, facing higher virus exposure [6] with this population accounting for around 21%, including 20% among nursing and support staff and 44% among medical staff [6, 7]. This risk increased stress and anxiety among HCWs, especially regarding their own safety and that of their families [8, 9].

While HCWs have been widely recognised as being at elevated risk of COVID‐19 infection due to occupational exposure, there is limited data on the transmission and health outcomes among their household members, particularly within minority ethnic groups. Emerging evidence suggests that living in multigenerational households (more common among ethnic minority communities) may contribute to increased COVID‐19 mortality risk. For example, Nafilyan et al. [10] found that elderly individuals from South Asian backgrounds living in multigenerational households had a significantly higher risk of COVID‐19 death compared to those living with other older adults. However, comprehensive data on second‐hand exposure from HCWs to their household members remains scarce. This gap underscores the need for further research to understand the indirect risks faced by families of frontline workers, especially in ethnically diverse populations.

During the early pandemic, HCWs faced significant mental and physical health challenges. The British Medical Association (BMA) reported that many doctors experienced anxiety, depression, burnout and 65% reported fatigue and exhaustion [11]. Surveys also revealed widespread concerns about personal and family health, with many HCWs feeling at risk due to their clinical duties [5]. Similarly, a Royal College of Nursing (RCN) survey [12] showed that most nurses and midwives felt at risk and were deeply concerned about their household members’ well‐being. Additionally, increased workload, extended hours and self‐isolation due to potential exposure significantly contributed to stress, anxiety and depression among HCWs, impacting their mental health [13]. These findings highlight a clear link between HCWs’ roles, heightened risk of contracting COVID‐19 and effects on mental health. Additionally, anxiety extended beyond professional duties to concerns for their household members’ health.

Exploring the experiences of Black, Asian household members living with HCWs was crucial, as this vulnerable group faced unknown challenges. Data shows that Black and Asian communities were at higher risk of severe COVID‐19 impacts, even after adjusting for employment, living conditions and health [3]. Also, reports highlight disproportionately high COVID‐19 admissions and deaths among these groups [14, 15]. Therefore, this paper focuses specifically on Black and Asian communities, as they were most affected during the pandemic. The disparities and heightened vulnerabilities of Black and Asian groups highlight the need to examine their experiences to understand the challenges faced by these individuals during this health crisis. Conducted in 2021, this study offers insights into managing future pandemics for Black and Asian HCWs’ households.

## 2. Methods

This study employed interpretative phenomenological analysis (IPA) to explore the meanings and experiences of household members living with Black and Asian HCWs during the pandemic in the United Kingdom because Black and Asians were the most hit. IPA is a qualitative approach that seeks to understand how individuals make sense of their experiences through detailed, interpretation of personal narratives [16].

The aim was to uncover their unique perspectives. IPA was used to aid understanding of participants’ experiences by interpreting the meanings they ascribe to their experiences [16].

### 2.1. Eligibility Criteria

This study included participants with direct relevant experience related to its focus. Eligible participants were Black or Asian, lived with a Black or Asian HCW (must be a family member—genetic or extended), were proficient in English, had telephone and email access, and were 18 or older during the COVID‐19 pandemic. Excluded were non‐Black or Asian family members, those under 18, or individuals unable to speak or read English.

This study adopted a broad definition of HCWs, including professionals in health or care settings such as doctors, nurses, midwives, nursing associates, healthcare assistants and AHPs. Adult household members were selected as they could best share their experiences of living with Black and Asian HCWs during the pandemic. English fluency was required to ensure participants could understand the study, sign consent forms and take part in telephone interviews.

### 2.2. Recruitment Strategy

Data were collected between December 2020 and February 2021. Participants were recruited primarily through worship centres using a snowball sampling approach. This strategy was selected for its accessibility and cultural relevance to Black and Asian communities [17]. No recruitment occurred via healthcare services or HCWs themselves. Interested individuals contacted the researcher to receive study details. Consent was obtained via email. Participants suggested other eligible candidates, who were then invited to participate, with the option to decline without pressure or consequences.

Six participants were recruited, consistent with IPA’s idiographic approach, which prioritizes detailed, in‐depth analysis over larger samples. IPA emphasises understanding individual experiences, with the researcher employing a double hermeneutic approach to interpret participants’ experiences [16].

### 2.3. Data Collection

Semistructured telephone interviews were chosen for their cost‐efficiency, ability to reach diverse locations and convenience [18]. Video conferencing was considered, but issues like limited Internet access, privacy concerns and technological challenges were noted. Due to COVID‐19, this method minimised face‐to‐face contact, ensuring the safety of participants and the interviewer. While lacking nonverbal cues, participant engagement remained unaffected.

The interview guide was designed to elicit detailed narratives, following IPA methodology. Open‐ended questions encouraged comprehensive responses, with probes expanding participant answers. A heuristics framework ensured rich data collection. A pilot interview assessed question suitability; no changes were required, and it was included in the study. Participants were briefed, assured of confidentiality and reminded of anonymity. Interviews, conducted privately by the researcher (UJ), lasted 30–60 min. The researcher followed up to address any issues and confirmed participants’ comfort.

### 2.4. Data Analysis

The researcher (UJ) used a structured approach to systematically analyse the data [16], exploring the nuances and significance of Black and Asian household members’ experiences of living with Black and Asian HCWs during the pandemic. The process followed six IPA‐guided steps. First, audio‐recorded interviews were transcribed verbatim and thoroughly reviewed to understand participants’ experiences. The transcripts were iteratively reviewed for a comprehensive understanding. Then, key points related to the research questions were noted, using descriptive, linguistic and conceptual comments (see Supporting table 1). Emergent themes were identified by condensing notes and analysing data for connections. These themes were organised hierarchically into subordinate and superordinate categories (see Supporting table 2). Individual transcripts were then examined independently, setting aside prior knowledge. Cross‐case analysis followed, identifying master themes by exploring shared and unique meanings. Verbatim quotes illustrated themes, highlighting participants’ contexts and idiographic experiences during the COVID‐19 pandemic.

### 2.5. Reflexibility

Throughout the research process, reflexivity was actively maintained to ensure transparency and minimise researcher bias. The lead researcher (UJ) kept a reflexive journal to document personal reflections, assumptions and emotional responses during data collection and analysis. This practice helped to critically examine how the researcher’s positionality, as a member of the same ethnic community and with professional ties to healthcare, might influence interpretation. Regular peer debriefing sessions were also conducted with the research team (RK and MM) and other colleagues experienced in qualitative research to challenge emerging themes and interpretations, enhancing the credibility and trustworthiness of the findings.

## 3. Results

### 3.1. Study Population

The participants were aged between 22 and 60 years old. Most participants were from an African background, and one was from an Asian background. Four participants were educated at master’s degree level, and their occupations varied. Pseudonyms were used to refer to participants. Please see Table 1 (uploaded separately) for details.

**Table 1:** Summary of participants involved in the study—Participants’ names are pseudonyms.

Participant & pseudonym	Ethnicity	Sex	Occupation	Educational background	Role of HCW	Age
1‐Haj	Black‐African	Female	Housewife	Diploma	Medical Dr.—daughter Nurse—husband Respiratory physiologist—son	52
2‐Tobi	Black‐African	Female	Business owner	Masters	Midwife—daughter Registered nurse—Husband	52
3‐Tunde	Asian Indian	Male	Shop worker	Degree	Registered nurse—mother and sister are both nurses. The participant lives with both.	22
4‐Onny	Black‐African	Male	Social worker	Masters, PhD student	Registered nurse—wife	37
5‐Obe	Black‐African	Male	Self employed	Masters	Registered nurse—wife	55
6‐Rash pal	Black‐African	Male	Social worker	Masters, PhD student	Registered nurse—wife	36

**Figure 1:**
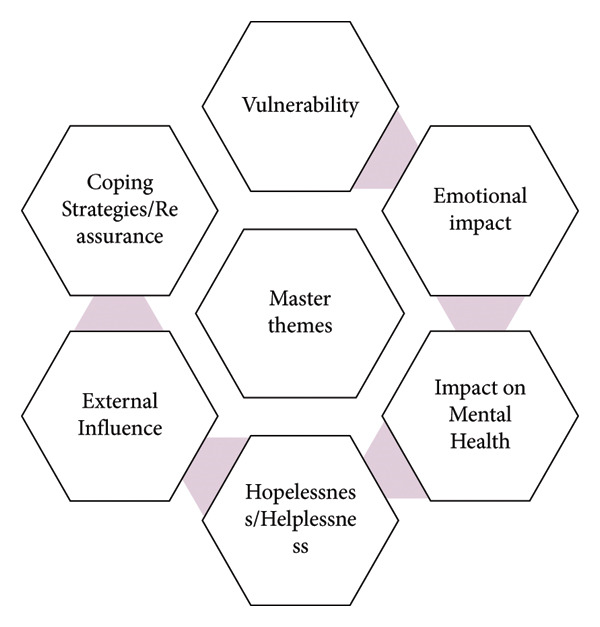
Master themes.

Following the cross‐case analysis of participants’ subordinate and superordinate themes, six master themes were developed—see Figure 1 (uploaded separately) for the master themes. Table 2 (uploaded separately) shows the frequency of cases within the master themes.

**Table 2:** Frequency of cases within master themes.

Master themes (across the cases)	N	Haj	Tobi	Tunde	Onny	RashPal	Obe
Vulnerability	6	✓	✓	✓	✓	✓	✓
Emotional impact	6	✓	✓	✓	✓	✓	✓
Impact on mental health	6	✓	✓	✓	✓	✓	✓
Coping Strategies	6	✓	✓	✓	✓	✓	✓
Hopelessness/helplessness	5	✓	✓	✓	✓	✓	
External Influence	5	✓	✓	✓	✓	✓	

The master themes are discussed below. Participants’ illustrations, which are drawn from the richest accounts of their transcripts are italicised. Some excerpts are written in shorthand and abbreviated, as the interviews were transcribed verbatim. The researcher’s comments are presented in regular text. BAME (written verbatim) in participants’ excerpts refer to Black, Asian and Minority Ethnic. All the participants had HCWs as household members who worked and directly cared COVID‐19 patients.

### 3.2. Theme 1: Vulnerability

The theme of vulnerability emerged as participants described feeling exposed during the COVID‐19 pandemic, influenced by their ethnic background and living with Black and Asian HCWs. Their narratives highlighted links between ethnicity, comorbidities, multigenerational living and the heightened COVID‐19 risk faced by HCWs. They expressed fears about the infection’s impact, believing little could mitigate its severity, a perception shaped by research and media reports.

Tobi (pseudonym) indicated that she believed she was at increased risk, simply because she belonged to an ethnic minority community:

“Yeah but being BAME, we are at risk. …Research has shown that we are at risk, and we can see that BAME have a lot of issues, you know…but being BAME alone, you are at risk all the same.”

For Onny, Black and Asian HCWs and household members were at increased risk of COVID‐19 and had higher risk because of their background:

“And it even became worse when the, when the news broke out that people from my background are more vulnerable than others and you see the casualties on the news and then the statistics say much about erm casualty from BAME ethnic. I mean from BAME group was very very devastating. I mean frustrating you know erm it’s just the feeling that what could happen next.”

### 3.3. Theme 2: Emotional Impact

The emotional impact of COVID‐19 on HCWs’ household members and the increased potential to contract the virus were expressed through fear.

Participants expressed fear in varied ways. For example, Haj shared her experience of how the impact of COVID‐19 on three of her household members who were involved in caring for COVID‐19 patients while living with her was frightening. This level of fear was demonstrated in her interview:

“Realising the severity which it affects black people, is very scary…So now having three dear ones, loved ones, fighting this infection which affects black severely more than any other race is like hell for me”. “It has affected me so much, most nights I can’t sleep, fear, panic, anxiety, and paranoia every time they go to work.”

Furthermore, Tunde expressed his concerns about his family members who were HCWs on the wards. He conveyed his fear, highlighting the heightened and unpredictable nature of COVID‐19 infection:

“Like having them working without knowing what to expect. Like, it’s fear. Normally like, they will get it. And it’s like, whether they will suffer really badly or not from it. Yes, it′s really worrying. And it doesn’t make me good at all.”

Tobi appeared to lose trust in her household members’ ability to protect themselves, as well as in health leaders’ capacity to safeguard Black and Asian HCWs at work. She empowered her household members to prioritise their safety. The quote below illustrates how this loss of trust stemmed from being classified as high risk for the impact of COVID‐19 and the inadequate protection of Black and Asian HCWs at work, despite their vulnerability, suggesting a sense of powerlessness.

“Because we have been classified as ‘High Risk’ right? So, in view of that, you can speak up and say no to some things and hopefully, they would erm, erm, let you be, and allocate you to somebody else who is not COVID positive.”

Furthermore, Haj felt unhappy as COVID‐19 caused physical separation and loss of intimacy between her and her husband. Her account suggests a burden resulting from the hidden nature of COVID‐19. She recalled the inability to touch, kiss or hug her husband on return from work:

“Having a husband who works with COVID patients, he comes back home, how are you supposed to sleep on the same bed with him? How can you touch your husband? How can you kiss the husband? Kissing is even out of it completely; you don’t even want to go near that. If you are scared already the person is infected, I’m scared of giving the usual welcome hug”‐

## 4. Theme 3: Impact on Mental Health

All participants described the precautions they took to prevent COVID‐19 from entering their homes, driven by its severe impact on Black and Asian communities.

In this interview extract, Haj explains the measures she implemented when her husband, son and daughter, who worked as a nurse, respiratory physiologist and doctor, returned home. Focused on safety, Haj’s approach became transactional, replacing the usual greetings between family members:

**“**All I hope is let them come back safely and before anybody gets back into the house when they get back from work, I’m always at the door; take off your shoes there. I’ve got the disinfectant tray spraying all the shoes, take off your clothes there. I’ve got new pairs for you to put on. Take of your shirt there. It’s just mentally affecting me and then I’m saying, please go straight to the bathroom”.

Similarly, Obe demonstrated the effect of precautions on his behaviour:

“Well… the other thing is that, subconsciously at times you want to take precautions so that other members of the household may not be infected and it may have a slight change on your behaviour—

Participants implemented measures to prevent COVID‐19 infection and demonstrated how the virus affected their behaviour and mental health. They placed significant importance on preventing infection within their homes due to its severe impact. It can be assumed that conflicts may arise within the household if any family member failed to comply with these precautionary measures.”

### 4.1. Theme 4: Coping Strategies: Peace of Mind and Support, Policy and Practice

Haj believed her fears, anxiety and mental health issues would be resolved if Black and Asian HCWs were allowed to stay at home with pay.

“What anybody can do to support me; leave my family at home for me and pay them because we need money…

That’s why I think if they ask them to stay at home, I will be happy. I will have peace of mind. The panicking will reduce. The anxiety and fear will go because what is creating the anxiety, and fear will go. If they stop going to work all that will stop, and then we can be happy again.”

For Onny, receiving reassurance about the protection of Black and Asian HCWs is essential. This reassurance not only alleviated concerns about their safety and well‐being but also fostered a sense of trust in the healthcare system. Onny expressed that it was crucial to feel confident that appropriate measures were in place to safeguard those who served on the front lines during the challenging times, particularly faced heightened risks. Onny suggested that this sense of security could ultimately contribute to mental health and job satisfaction for HCWs and their household members.

“Okay that your wife or your husband is going to the hospital, we are there to protect them, we are there to support them, we are there to support you, this and that if you require support or question at any time, number or email this, or you could chat with this, at least it should be there really.”

Tunde proposed transferring Black and Asian HCWs to non‐COVID‐19 wards to reduce their disproportionate risks. Aware of their heightened vulnerability due to social and health factors, he emphasised creating a safer work environment while maintaining patient care. His recommendations stress prioritizing the safety and well‐being of Black and Asian HCWs.

“I’m not sure how specifically, that can be helped. If there’s any way that the more vulnerable workers, maybe are not transferred to COVID positive wards, or maybe I’m not sure but a lot of people got redeployed and staff from different places maybe like I mentioned, that my mum got deployed, may be knowing she’s more vulnerable than others, The other people who are less vulnerable should be redeployed instead.”

However, Onny suggested policies are needed to protect Black and Asian communities:

“How? Like I said before, you know if, if they could actually, like policies, there are different policies you know that could protect them really, that would give them more voice.”

Also, participants demonstrated the importance of faith during the pandemic. They felt that the presence of God in their lives served as a reassurance to their survival. Tobi said she called on God during the COVID‐19 pandemic because her faith gave her reassurance:

“Calling God means satisfaction, means I have relationship with him”. “Anytime I call Him, I feel reassured, I feel satisfied, I feel calm”.

Onny expressed similar views which reinforce the importance of faith in these communities:

“We can as humans do all we need to do to help ourselves. We just need to rely on God especially in this our situation right, nothing makes any sense. If you think about it very well, nothing makes any sense. When the pandemic broke out, nobody knew that the BAME community were, were at risk, cause everyone going out but as more light was shown on the virus we found out that BAME were more vulnerable”.

In summary, participants’ narratives depict hope, and a sense of reliance on religious faith for safety. Faith played a role in participants’ survival during the COVID‐19 pandemic. Faith, being participants’ source of reassurance and hope is unsurprising as they were recruited from a worship centre.

### 4.2. Theme 5: Hopelessness/Powerless and Helplessness

Respondents felt hopeless, helpless and powerless due to insufficient protective measures for Black and Asian HCWs, the severe impact of COVID‐19 and higher mortality risks in minority groups compared to nonminority groups. They also expressed distress over being unable to protect family members from workplace exposure due to financial pressures from job loss.

Haj believed that being from a Black or Asian background heightened vulnerability to the virus and reduced survival chances, deepening her despair:

“So that kind of creates more panic and pressure and if a White person gets it, they have more chances of recovering. But if a black person gets it, they have no chance of recovery from it, less chance let me not say no chance. And for those who recover it’s more problems. It leaves them with long term disability so that is very disheartening. Where is the hope”? Now, what hope is there? It means once a black person catches the virus we are in trouble, we are done. The family is doomed.”

“We don’t have any power, what can we stop? We can’t stop. I can’t stop my husband or children from going to work and the virus is there, and they are working with the virus. Is like they are in the battle field, and I can’t fight it, I can’t fight it or rescue them, I am vulnerable like them.”

However, Tunde expressed feeling helpless because he perceived no effective measures were in place to reduce the risk of infection for Black and Asian HCWs in their workplace:

“There is no, there is no measures in place to kind of like prevent them because there are increased chances of BAME people catching Coronavirus. But there is nothing in place to lessen that likelihood, if that makes sense. Okay.”

Tobi’s husband is an agency nurse, and her daughter is a midwife. Tobi raised her concerns as she was unable to stop her husband and daughter from working due to their increased risk. She was worried about her husband contracting COVID‐19 and felt helpless as his unpaid leave would affect household finances, leaving her deeply worried:

“I wouldn’t say they shouldn’t go to work because left for me he, okay, if we say because they are at risk, what can we do? You cannot say they shouldn’t go to work, can you.”

“For example, if my husband is ill now he is still gonna go to work because you have bills to pay. You know, there’s lot of bills to pay. He is not with the NHS.”

#### 4.2.1. Theme 6: External Influence—The Media

The media played a significant role in shaping perceptions of the COVID‐19 pandemic. Participants noted both positive and negative influences.

For instance, Onny shared that media coverage heightened his awareness of his vulnerability as someone from a Black and Asian background. It provided insight into his group’s susceptibility and the severe impact of COVID‐19.

“Yeah, I think the news is just there to enlighten me, to keep me informed on what is going on around the world. My views, it just makes me to be more aware of how vulnerable we all are, as people and also as BAME you know individuals, you know, it yeah, it makes me to be more aware of that.”

For Tobi, the news report about infections and the deaths among Black and Asian groups cause fear:

“A lot of them lost their lives. A lot of them were infected. So, that alone is scary you know. Scary days, everyday listening to news, seeing the same things and you are thinking Oh my God, I have got, these are my people, they are out there.”

## 5. Discussion

This study offered valuable insights into the experiences of household members living with Black and Asian HCWs during the COVID‐19 pandemic in the United Kingdom. Themes such as reassurance, vulnerability, hopelessness and helplessness are specific to the COVID‐19 context and the experiences of Black and Asian HCW household members. However, some aspects align with prior literature on past pandemics and communicable diseases, including fear [19–21]. These fears varied, such as mothers fearing for sick children during Uganda’s 2000–2001 Ebola outbreak and caregivers reporting beliefs that visitors left their homes with Ebola [20]. Participants in this study feared COVID‐19 contagion due to the heightened risk faced by Black and Asian HCWs. This aligns with prior research on fear during disease outbreaks [20, 21], where HCWs feared infection and spreading the virus to household members. However, fear in this study varied, driven by concerns about infection and its severe impact on Black and Asian communities, highlighting the emotional complexity of this context.

Participants experienced significant sadness, due to concerns about inadequate protection for Black and Asian HCWs, fear of death and loneliness. Although the fear of death was a common theme during the pandemic in similar studies [22, 23], the fear of death and concerns about inadequate protection in Black and Asian group was heightened due to systemic disparities, their increased vulnerability and the severe impact of COVID‐19. The sadness and loneliness tied to potential loss were unique to household members of Black and Asian HCWs. They felt doubly vulnerable: at risk of infection from living with HCWs and impacted by COVID‐19’s disproportionate toll on ethnic minority communities. This theme underscores the complex connection between identity and existential fears, which is unique to this study.

The pandemic led participants to adopt precautionary measures at home, influenced by their perception of risk and severity, consistent with the Health Belief Model [24]. This model outlines six constructs predicting behaviour: risk susceptibility, severity, benefits, barriers, self‐efficacy and cues to action—reminders of potential health issues [24–26]. Glanz and Bishop [27] emphasise the importance of families, socioeconomic status, demographics, culture and social relationships as key influences on behaviour change and the maintenance of healthy behaviour. In this study, participants from Black and ethnic minority backgrounds, along with their perceptions of susceptibility and the severe impacts of COVID‐19, were significant cues prompting precautionary actions. However, these protective measures also contributed to stress and anxiety, highlighting the delicate balance between safeguarding health and managing psychological strain during the pandemic.

Household members faced various losses, including intimacy and physical contact, due to the pandemic. This aligns with prior studies on separation during communicable diseases like HIV and *tuberculosis* [19, 28]. Participants in this study feared not only health risks and vulnerability from living with HCWs but also potential livelihood loss if their partner fell ill or died. At the early time, many viewed COVID‐19 as a death sentence, heightening anxiety. Household members also felt powerless against the virus, despite government measures, citing its power and persistent spread as a source of fear.

Anxiety among household varied, stemmed from uncertainties about COVID‐19’s impact on Black and Asian HCWs and heightened risks for ethnic minorities. Household members of HCWs in the general public also experienced anxiety during the COVID‐19 pandemic. However, the anxiety among these groups of people concerned their health workers’ work, physical health, hearing about traumatic experiences including organisations’ inability to meet their needs and their daily lives being disrupted due to taking family responsibilities [29, 30]. Furthermore, media reports significantly influenced information during the pandemic. Matthews et al. [31] note that health news sourcing becomes complex during crises, disrupting normal gatekeeping and narrowing sources, potentially leading to inaccuracies. Similarly, while COVID‐19 media coverage improved public adherence to health measures in Sweden and the United States, some outlets engaged in biased reporting, making misinformation a topic of research and debate [32].

News reports on COVID‐19’s severe impact on Black and Asian groups had positive and negative effects on respondents. They increased awareness of the virus but also heightened fear and anxiety about its impact on these communities. Similarly, a review in Hong Kong found that supportive measures reduced negative emotions, while public comments on government actions reflected negativity [33]. The study suggested that neutral media tones could help mitigate public anger.

In this study, seeking reassurance and adopting increased precautionary measures were coping strategies for anxiety. Participants felt helpless about being unable to prevent HCWs from working despite the risks. This powerlessness and lack of control deepened their hopelessness. Psychological and emotional support emerged as essential for coping while living with Black or Asian HCWs during the pandemic.

### 5.1. Strengths and Limitations

This study offers valuable insights into the experiences of household members of Black and Asian HCWs during the COVID‐19 pandemic. By highlighting the unique challenges faced, it underscores the emotional and social impact of the pandemic on these communities and the broader implications for HCWs. However, the findings should be considered alongside certain limitations.

First, the recruitment strategy, primarily through worship centres, may have introduced selection bias, favouring participants with strong faith‐based coping mechanisms and potentially excluding secular perspectives.

Second, although the study aimed to include a diverse sample of minority ethnic groups, the final composition was predominantly Black African. This limits the breadth of perspectives from other ethnic communities, particularly South Asian groups, and may affect the generalisability of the findings across the wider minority ethnic population.

Additionally, conducting interviews by telephone restricted the ability to observe non‐verbal cues, which may have affected the depth of emotional insight. The small sample size (*n* = 6), while consistent with IPA methodology, further limits generalisability. The sample also skewed towards educated individuals, potentially excluding the experiences of less‐educated household members who may face different challenges.

Finally, while reflexivity was maintained throughout the research process, the lead researcher’s positionality and the subjective nature of IPA may have influenced interpretation.

## 6. Summary

This study uncovered the complexities, vulnerabilities and coping strategies of household members of Black and Asian HCWs, providing insights into their needs and experiences. Respondents’ idiographic accounts revealed that external factors and living with HCWs, significantly affected their emotional and mental well‐being. Participants felt powerless, hopeless and vulnerable due to their backgrounds, prompting them to seek reassurance. The findings offer recommendations for healthcare policies to support Black and Asian HCWs and identify necessary services for their family members.

## 7. Recommendations and Practical Implications

This study highlights the importance of creating platforms for Black and Asian HCWs to voice concerns and participate in decision‐making, enhancing their security and addressing unique challenges. Support for their households is equally vital to foster a supportive environment. Addressing protective measures for these HCWs is crucial for their confidence, well‐being and to reassure their families and communities. Tailored seminars, educational programs and accessible resources can help alleviate fears and provide accurate information.

Our findings highlight this group’s experiences and provide a foundation for tailored interventions and support systems. The focus on emotional disturbances and nuanced experiences can inform future healthcare and community support policies.

## Ethics Statement

This study was approved by the University of Wolverhampton ethics committee, with reference 1120UJUOWHEA.

## Consent

Consent to publish has been obtained from participants.

## Disclosure

Preregistration of studies plan. The research was not preregistered with an independent, institutional registry. Preregistration of analysis plan. The research was not preregistered with an analysis plan in an independent, institutional registry.

## Conflicts of Interest

The authors declare no conflicts of interest.

## Author Contributions

Ufuomanefe Jones, Doctoral researcher, contributed to conceptualisation, literature review, formulation of research questions and objectives including interview guide, methodology, data collection, formal data analysis, original and all writing, and research. Amanda Rodrigues Amorim Adegboye assisted with framing the material into a manuscript format and editing the material and reflection and interpretation of the findings. Ranjit Khutan, Doctoral supervisor, contributed to the development of the research question, methodological rigour, critical and intellectual input during data analysis, and interpretation phases including validation. As my supervisor, he was responsible for the integrity and accuracy of the work, ensuring adherence to ethical standards. His active involvement extended to reviewing and revising the manuscript, improving its clarity and quality. Moses Murandu, Doctoral supervisor, contributed to the development of the research question and methodological rigour critical and intellectual input during data analysis and interpretation phases. As my supervisor, he was responsible for the integrity and accuracy of the work, ensuring adherence to ethical standards. His active involvement extended to reviewing and revising the manuscript, improving its clarity and quality.

## Funding

This research did not receive any specific grant from funding agencies in public, commercial, or not‐for‐profit sectors.

## Supporting Information

Supporting table‐S1 Sample showing a part of a transcript and emergent themes (extract) Supporting table S2 Sample of steps of analysis for Tobi (extract). These supporting materials have been uploaded separately.

## Supporting information


**Supporting Information** Additional supporting information can be found online in the Supporting Information section.

## Data Availability

The data that support the findings of this study are openly available in University of Wolverhampton at https://hdl.handle.net/2436/625108.
