# Supplementary material for: Family Experiences of Living With Frontline Healthcare Workers During the COVID‐19 Pandemic in the United Kingdom: Perspectives From the Black and Asian Community
Source: Interdiscip Perspect Infect Dis. 2026 Jan 13;2026:5200232. doi: 10.1155/ipid/5200232 (PMC12797149; doi:10.1155/ipid/5200232)
Supplement: Supplementary file 1 — Supporting Information Additional supporting information can be found online in the Supporting Information section. [file IPID-2026-5200232-s001.docx]

**Supplementary 1**

**Sample of a part of a transcript and emergent themes (extract)**

**Key**- Adopted from Smith *et al.,* (2009) pg 84

| Descriptive comments | Focused on describing the content of what the participant has said, the subject of the talk within the transcript (normal text) |
| --- | --- |
| Linguistic comments | Focused upon exploring the specific use of language by the participant (Italic) |
| Conceptual comments | Focused on engaging at a more interrogative and conceptual level (underlined) |

| **Transcript** | **Exploratory remarks** | **Emergent themes** |
| --- | --- | --- |

| 1. I: Okay thank you. Can you tell me about your experiences of living with a BAME healthcare worker during COVID 19 pandemic? 2. R: Ah it’s a long story, because ehm you know, we are black like I said, we are originally from Nigeria, so we black Africans and everything we hear on the news is the severity of how this COVID affects blacks, more than the white, ehm the white people. So now having 3 dear ones, loved ones, fighting this infection which affects black severely more than any other race **is like hell** for me. It has affected me so much, most nights I can’t sleep, fear, panic, anxiety, and paranoia every time they go to work; I’m a full time housewife, so I stay home I do the cleaning, the cooking and all those things. I clean and clean nonstop, its affecting my mental health seriously. All is the fear of ehm disinfecting the virus or trying to get rid of the viruses if any has been brought into the house cause if eventually if they get infected, I’m going to get infected, and all of us in the house will get infected and realizing the severity which it affects black people, is very scary. **And then every time they go to work before they live, I’m always warning and advising and begging and pleading please be extra careful, please be very safe, please wash your hand 100 times**, please don’t take off your mask, please make sure anything you touch you wash your hand I’m just so anxious and restless, **is like I should just follow them to work, so I can look after them so they don’t get it.** As if I can actually stop them from getting the virus that is how **anxious and worried and panic** I get. And then I’m always calling even when they are at work every now and then; what are you doing now, are you okay? Have you washed your hands? Are you wearing your mask? Please don’t speak to anybody I don’t think I’m normal anymore to be honest. I’m just so so much affected it’s scary cause I can imagine my husband for example getting it, he is almost 60, and may be dying from it, or **becoming paralyzed from if or having some complications God forbid**. It’s so scary or my precious children you know, especially the one that is a medical doctor, she is placed in the ward where all she does is treating this COVID patients everyday **I’m like almost crying when she is going to work.** I’m always in tears everyday she’s going to work **I’m so worried I’m so scared I always panic I’m always in tears everyday** .She keeps saying don’t worry mum we will be fine, we are taking precautions **pray for us**, **but then my mind is never at rest**. I’m worried my blood pressure have shot up significantly since this pandemic started**, I never use to be a hypertensive patient but from the lack of sleep from worrying and thinking have given me high blood pressure and now I’m on high blood pressure medication.** **I don’t sleep most nights, now I’m on tablets to sleep in the night**, **is just beyond description**. I don’t know where to start from its not something that can be described. **I’m so emotionally involved**, **I’m so psychologically tensed.** **It’s just killing completely.** It’s like I’m living but I’m actually dead’. I’m worried. **I’m not living life**, **I’m not enjoying life.** **I’m just like monitoring my family, begging, pleading, and supporting**. **I’m not living life.** **I’ve stopped living life since 2019 December** when this pandemic thing started and got worse and then when it got to 2020 and we have all been locked down. **Basically we are not living life.** **I’m not living life** because all I’m doing instead of thinking ahead doing things that I love to do nothing. **My life is just at a standstill.**  All I hope is let them come back safely and **before anybody gets back into the house when they get back from work, I’m always at the door; take off your shoes there**, **I’ve got the disinfectant tray spraying all the shoes**, take off your clothes there. I’ve got new pairs for you to put on. Take of your shirt there.. ..it’s just mentally affecting me and then I’m saying, please go straight to the bathroom. **At a point it’s almost breaking up my marriage**, because I no longer sleep in the same room with my husband because I’m scared of the virus. I’m like no no no **we can’t sleep on the same bed**. You are working in the hospital, I’m at home, I don’t want the virus. So you can see how much it has affected the family. **This is just some of the things I can mention. Honestly, I can talk from now till tomorrow, telling you how this pandemic has affected my life and I will not get finished**. I can keep telling you and telling you. We don’t do anything intimate that requires physical contact, just for the fear of this virus.. Pause | Hesitation- (ehm, you know).  She appears to have knowledge of the severe impact of covid-19 on BAME population.  The involvement of 3 BAME HCWs in the care of COVID-19 patients is frightening.  *Metaphor- fighting this infection, signifying the strength of COVID-19*.  *“Is like hell” shows the degree of anxiety, fear and emotional instability*.  BAME HCWs going to work causing loss of sleep, panic and having impact on her mental health.  Carrying out precautions to prevent infection in her home.  Repetition- I clean, I clean nonstop- showing impact of fear of contracting COVID-19.  Is there intense fear here?- very scary  She seems worried about HCWs leaving home for work due to fear of being infected, she is being extra careful in relation to protecting BAME HCWs and self from COVID-19 infection  Elements of loss of self and feeling vulnerable. Seems she has lost control of emotions.  Overwhelmed with fear, panic and anxiety  There appears to be loss of confidence on the ability of BAME HCWs to protect themselves. There seems to be a level of insecurity  Is there sense of insecurity?  COVID-19 has changed self  Loss of identity  Repetition “I am so, so” showing inability to articulate words due high level of fear and worry  Fear of death of loved one due to age and severe impact of COVID-19  Fear of unpredictable course of COVID-19.  God forbid- Nigerian term meaning “may God prevent bad things from happening”  Seems to express feeling of BAME HCWs being vulnerable at work.  Feeling of frustration, sorrow and sadness with BAME HCWs going to work  Expression of sadness  Overwhelmed with worry leading to loss of sleep. Significant effect on blood pressure since onset of COVID-19 pandemic.  Seems not to relent on worries despite reassurance from HCW and request for prayers.  Seems to have family bond  BAME HCW’s request for prayers suggests the family seem to believe in prayers.  Sense of vulnerability and loss of control.  Seems not to have any options. Helpless  Is there an element of frustration?  Is there solace in God?  Seems to be overwhelmed with emotions  *Representation-it’s just killing completely, suggesting mental torture and frustration*  *Intense sense of grief and sadness*  *Not living life, stopped living life- meaning loss of self -worth, image of new self that is less able and hopeless*  *Not enjoying life- demonstrating despair and sense of grief*  *Stopped living life- hopelessness*  Emphasizing precautions and worries  Prevention of suspected infection  Fear of COVID-19 infection and change in behaviour affecting relationship and intimacy  Loss of intimacy  *Rejection-no no no suggests emphasis on sleeping apart*  Distancing and separation  Unending description of the negative impact of COVID-19 on herself and family.  Loss of intimacy due to fear of COVID-19 infection | Fear of COVID-19 infection  COVID-19 is severe  Anxiety and emotional instability  Precautions to prevent COVID-19 infection  Impact on COVID-19 mental health  Fear of COVID-19 infection  Protecting BAME HCWs and self  Fear, panic and anxiety  Loss of control of emotions  Loss of self  Vulnerable  Overwhelmed with fear  Sense of insecurity  COVID-19 changed self  Loss of identity  Fear and worry  Fear of death  Unpredictable course of COVID-19  Sadness  Sorrow  Frustration  Sadness  Vulnerability  Believe in prayers  Solace in prayers  Helpless  Loss of control  Frustration  Grief  Loss of self- imagine  Hopelessness  Frustration  Mental torture  COVID-19 changes self  Separation  Loss of intimacy  Rejection  Loss of intimacy |
| --- | --- | --- |
| 1. I: Thank you very much but if you don’t, if you don’t mind can I take you back a little bit, and then we just walk through. Now you did mention ehm you talked about the news right? And the severity of the pandemic ehm what do you mean you know, about the news what do you mean by that, talking about the severity of the pandemic? 2. R: You know the media is one thing, social media is another thing, and the world is now a global village, this is technology, so information pass freely you get a lot of text messages from friends, groups, WhatsApp groups or twitter all this things. And it’s all very **scary.** What we see is the virus affecting black people and how much it destroys and kills them or paralyse them and make them not to be able to function again, maybe end up on wheelchair, heart failure and all this problems . The media itself even the NHS, the Government they keep raising alarm that black people are dying more. **They keep giving us statistics black people are dying more they give it in America, they give it here, everything is on the news**. **More black people are dying from is, it affects black people more for whatever reasons, black people need to do risk assessment, I don’t know what kind of virus takes more of blacks than white**. I just don’t understand, but we could see statistics and the government keep showing it**. The NHS keep announcing it in the news whether BBC, whether sky, whether CNN, they have been giving us the figures, particularly in 2020 during the first lockdown. I think that was around April May, June, they keep showing how many BAME people have died from this**. **They keep giving some justification why it’s affecting blacks more or whatever is the reason, we are black and it means we have higher risk, and kind of create more fear, more panic, more mental problem.** 3. I: Okay 4. R: In short my blood pressure as well, and there is nothing I can do than just worrying, crying every day. | There seems to be media and social group involvement causing more fear and anxiety in regards to the severe negative impact of COVID-19 on BAME population.  She appears to be knowledgeable about the impact and complications of COVID-19 on BAME population  Questioning the nature of COVID-19    Is COVID-19 discriminative?  Displaying statistics of death of BAME population by the media/ government seems to worsen fear of death of BAME population from COVID-19  She seems helpless, hopeless and vulnerable and frustrated  Losing control | COVID-19 causing death of BAME population  Fear  Anxiety  Media and social media causing more fear and anxiety  COVID-19 causing death  COVID-19 is ? discriminatory  Losing control  Hopeless  Helpless  Vulnerable  Frustrated |
| 1. I: Okay, so how do, you know this experience and the news and the media, how does it make you feel? How do you feel with all this news and information coming up? 2. R: **That’s exactly what I’m saying, they make you become scared, and they make you fear** 3. I: Okay 4. R: **They give you no hope it’s very hopeless they just create more paranoia in you and it’s affecting my mental health** **cause every day I listen to the news, I hear more blacks are dying than whites, and more blacks**… then it also makes you feel like there is a discriminative thing here, because how should the virus know blacks from white? They give excuse; the body make up, the lack of vitamin D in blacks and those are medical excuses they give, but the fact remains that we are blacks, and we are the victims and that kind of create more problems for me, more fear. Pause | News and media causing fear  Media causing hopelessness and more mental health issues regarding information on the death of more non – White population.  *Questioning the nature of COVID-19*  She seems to feel that COVID-19 is a discriminatory disease.  Is she having a feeling that information from media is unreal?  Emphasis on non-Whites being victims of death from COVID-19  Emphasis on fear of BAME death from COVID-19 | Fear  Hopeless  Vulnerable  Non-whites victims of death from COVID-19  from Media information causing increased fear  COVID-19 is discriminatory |
| 1. I: Thank you. So you did mention you have no hope, what do you mean no hope? 2. R: **Well if the government themselves and all this people are saying that the virus is affecting blacks more, all the social media are saying that, the entire news, the WhatsApp messages, and individuals, everybody saying its affecting blacks more and there is nothing that can be done**, it’s because of other things in the blacks blood maybe lack of vitamin D, lack of whatever. Now what hope is there**? It means once a black person catches the virus we are in trouble, we are done. The family is doomed.** So that kind of creates more panic and pressure and if a white person gets it, they have more chances of recovering, but if a black person gets it, they have no chance of recovery from it less chance let me not say no chance. And for those who recover it’s more problems. It leaves them with long term disability so that is very disheartening. Where is the hope? **Hope would have been something like if the media are saying it affects everyone equally and they is may be 50 percent chance of healing, may be 90 percent chance of healing or then that will be a flat equality form** but when they are saying it is affecting more blacks, and I’m black and they are saying they is less chances for the blacks to recover from it, and that is me black than the white counterpart, so I’m seeing the white, okay; you can get it, you are still better off, before you get it 10 or 12 blacks would have gotten it, your chances of getting it as a white is less, then as a white you have more chances of recovering, you have hope, you have hope not to get it, you have hope to recover if you get it.. But here is a black you have no hope, your chances of getting it is high 100 percent hopeless number one. Hopeless number two recovering from it is almost 0 hopeless number three, no hope. 3. I: Thank you. Thank you very much. So you did mention earlier that you have 3 loved ones you know who go to hospital to work, and you did say that for you, life is like hell, what do you mean hell? 4. R: Is the feeling isn’t it? Your worrying, the panicking, the things internally and even physically that I go through when they go to work. The mental torture, the fear you know; oh God they are going again, take care of them, protect them, don’t let any evil happen to them don’t let them catch this disease that is it, it’s just the fear that is it, it’s like not living life, it’s a different stuff from my normal self before the virus in fact before the virus, we were happy people we think positively; so what are I doing today, going for a walk, I’m going shopping.. The life now is completely different it’s no longer happy, it’s full of thinking and worrying, and sadness panicking and depressing News and everything. | She feels hopeless due to information from the government, News and social media describing more death of non-White population from COVID-19.  She feels contracting COVID-19 by non-White population is a doom/ death sentence  Does COVID-19 always cause death in non-White population?  Is COVID-19 infection a death sentence in non-White population?  She is expressing sadness from the long term complications arising from COVID-19 infection in non-White population.  *Questioning the reality or existence of hope “where is hope?”*  She seems to feel there will be hope if COVID-19 is described by the media as affecting all population equally, or chances of survival are the same for Whites and non –Whites.  The more vulnerability of non-Whites contracting the virus including less chances of survival compared causes hopelessness.  Emphasis on the chances of more survival of Whites from COVID-19  Hopelessness as there is increased rate of infection and reduced rate of survival by non-White population  Unseen and indescribable feelings and frustration when BAME HCWs go to work  She believes in God-Calling on God to take care and protect loved ones from evil and from getting COVID-19 infection.  Fear of loved ones contracting COVID-19 at work.  *Life is not the same since emergence of COVID-19-“ it’s like not living life, it’s a different life from her normal self before the virus”*  They were a happy family before the emergence of COVID-19  COVID-19 has caused sadness, panic and worry. | COVID-19 infection in non—White is a death sentence  Non-White population is safer with COVID-19 infection  Fear of death  Panic  COVID-19 causing doom  Sadness due to perceived complications from COVID-19 infection  Hopelessness  Hopelessness  Loss of future  Believes in God for protection from COVID-19 infection  COVID-19 changes self  Loss of identity  Loss of self  Sadness  Worry |

**Supplementary material 2**

Table- Sample of steps of analysis for Tobi (extract)

| Original Transcript | Exploratory comments | Emergent themes | Subordinate themes | Superordinate themes |
| --- | --- | --- | --- | --- |
| Emm.., my experiences is kind of emm..,a bit of fearful, you know, emm..,so since they are seeing patient on a daily basis at work, both my daughter, my husband and I feel some kind of emm.., at risk in term of being vulnerable, you know. They are the one treating the, the patient with COVID and they come back home, I feel kind of scared, I feel afraid of because I always think, maybe, ‘O my God’! as they have gone out there, they have, they are at the risk of contracting it before giving it to me at home | *Repetition erm erm-Seems trying to articulate words or finding difficulty putting words together*  Appears to find difficulty articulating words due to fear.  She is fearful and feels vulnerable as a result of BAME HCWs being at work facing COVID-19 patients every day.  She also feels scared and at risk in relation to the possibility of BAME HCWs bringing COVID-19 infection to her home | Fear BAME HCWs are bringing COVID-19 to the home | Fear | Emotional disturbance |
| yeah but being BAME, we are at risk. Research has shown that we are at risk. And we can see that BAME will have a lot of issues, you know. **Though I dont have, emm.., I wouldn’t say I have any medical. I don’t have any medical history that could at least make me at risk**. But being BAME is being BAME alone, you are at risk all the same. Yeah. Then if you now have more health issues which I don’t have. Although, it still makes me more anxious because am BAME, number one, and because my BAME family is out there, they go and come back make me anxious, that alone | Although she has no health risk factors, she remains anxious being BAME, and as BAME HCWs are out there at work | Being BAME is being BAME  At increased risk of COVID-19 infection irrespective of existing comorbidities | Being from BAME background | Vulnerability |
| I would think, number one, if they can exclude BAME people from looking after COVID positive patients. I don’t know how possible that gonna be. But it is, I don’t know; but that works, I think. Because that’s the way they could you know emm.,kind of prevent them from having it. If they, at least at work, they can ask them, what would you like to do? They can give them the choices, you understand | She thinks BAME HCWs should be protected from contracting COVID-19 at work by being prevented from caring for COVID-19 positive patients or they should be given choices of not caring for those patients | BAME HCWS should be protected at work | Relief | Peace of mind/Policy |
